# Influence of Individual Surgeon Volume on Oncological Outcome of Colorectal Cancer Surgery

**DOI:** 10.1155/2015/464570

**Published:** 2015-09-03

**Authors:** Marleen Buurma, Hidde M. Kroon, Marlies S. Reimers, Peter A. Neijenhuis

**Affiliations:** ^1^Department of Surgery, Alrijne Hospital, Location Leiderdorp, Simon Smitweg 1, 2353 GA Leiderdorp, Netherlands; ^2^Department of Surgery, Leiden University Medical Center, Albinusdreef 2, 2333 ZA Leiden, Netherlands

## Abstract

*Background*. Surgery performed by a high-volume surgeon improves short-term outcomes. However, not much is known about long-term effects. Therefore we performed the current study to evaluate the impact of high-volume colorectal surgeons on survival. *Methods*. We conducted a retrospective analysis of our prospectively collected colorectal cancer database between 2004 and 2011. Patients were divided into two groups: operated on by a high-volume surgeon (>25 cases/year) or by a low-volume surgeon (<25 cases/year). Perioperative data were collected as well as follow-up, recurrence rates, and survival data. *Results*. 774 patients underwent resection for colorectal malignancies. Thirteen low-volume surgeons operated on 453 patients and 4 high-volume surgeons operated on 321 patients. Groups showed an equal distribution for preoperative characteristics, except a higher ASA-classification in the low-volume group. A high-volume surgeon proved to be an independent prognostic factor for disease-free survival in the multivariate analysis (*P* = 0.04). Although overall survival did show a significant difference in the univariate analysis (*P* < 0.001) it failed to reach statistical significance in the multivariate analysis (*P* = 0.09). *Conclusions*. In our study, a higher number of colorectal cases performed per surgeon were associated with longer disease-free survival. Implementing high-volume surgery results in improved long-term outcome following colorectal cancer.

## 1. Introduction

The incidence of colorectal cancer is one of the highest malignancies [1]. In Netherlands alone, colorectal cancer is diagnosed in 12,000 patients annually and it is the second most frequent cause of death due to malignancies [2].

In an effort to improve the standard of care for these patients, new techniques have been introduced over the years, such as laparoscopy and TME surgery [3, 4]. Recently, much attention has been given to patient volume of both the hospital and the individual surgeon [5]. Publications have shown that a high-volume surgeon operating in a high-volume hospital leads to an improved short-term outcome such as a lower number of adverse events, shorter hospital-stay, lower postoperative mortality, and cost reduction [6]. Furthermore, a number of studies have reported increased long-term survival when patients are treated in high-volume centers [7]. However, the relationship between operative volume of the surgeon and long-term outcome remains unclear. We therefore conducted the current study to evaluate survival rates of patients with colorectal cancer following a procedure performed by high-volume surgeons compared to low-volume surgeons.

## 2. Materials and Methods

The Rijnland Hospital is a teaching hospital in Leiderdorp, Netherlands, serving approximately 200,000 people. For the current study a retrospective analysis was conducted from our prospectively collected database including all colorectal cancer patients who underwent surgery in our hospital between 2004 and 2011. Informed consent was obtained from all patients.

Eight hundred and twenty-four patients underwent a colorectal procedure between 2004 and 2011. For our study we used the same inclusion criteria as the national web-based registry for the surgical treatment of colorectal cancer in Netherlands: the Dutch Surgical Colorectal Audit (DSCA) [8]. Patients were excluded in case the procedure was performed for metastatic disease following previous surgery (*n* = 13), in case the primary tumor could not be resected (*n* = 9), or when the pathology report showed a different type of tumor than an adenocarcinoma (*n* = 28). After applying these exclusion criteria our study population of 774 patients consisted of a homogenous cohort.

In order to qualify as a high-volume surgeon a cut-off point of 25 colorectal resections per year, averaged over the study period, was chosen based on recent studies [6, 9–13]. Taking this criterion into account for our analysis, 13 low-volume surgeons operated on 453 patients and four high-volume surgeons operated on 321 patients. All 17 surgeons were senior attending surgeons in our hospital. Perioperatively, all patients received equal care using the colorectal enhanced recovery after surgery (ERAS) protocol [14, 15].

The data collected in our database were the patient characteristics, including the American Society of Anesthesiology- (ASA-) classification [16]; the intraoperative data (high-volume surgeon versus low-volume surgeon); and the postoperative data, including the TNM-stage [17], resection margins, length of hospital-stay, and adverse events. In case of an adverse event, the type (surgical or nonsurgical) and the severity were recorded according to Netherlands' Society of Surgery standard [18, 19].

Follow-up took place in our hospital according to Netherlands' Society of Surgery protocol [20]. This protocol dictates that patients are seen in the hospital for follow-up by an attending surgeon every 4 months for the first 2 years and every 6 months for the years after, with a minimum of 5-year follow-up. In the current study patients were followed up for a minimum of 3 years. At each visit, an ultrasound and CEA levels were performed.

Survival data were collected from our in-hospital patient records. Also the IKNL (Integral Cancer Centre Netherlands) was consulted in case a patient deceased, which provided us with the date and cause of death. For some patients the follow-up did not take place in our hospital, mostly due to relocation of the patient. In those cases we consulted the general practitioner and the hospital where the follow-up was taking place for survival data. It was possible to evaluate the 5-year disease-free survival (DFS) of 761 patients (13 patients lost to follow-up: 8 low-volume and 5 high-volume) and overall survival (OS) of 772 patients (3 low-volume patients were lost to follow-up).

### 2.1. Statistical Analysis

For assistance with the statistical analysis, the Department of Statistics in our hospital and the Leiden University Medical Center were consulted. Comparisons were made between the high-volume and the low-volume group for all variables: perioperative characteristics, disease-free survival, and overall survival. All statistical analyses were performed using SPSS version 20. The *χ* ^2^-test and the independent sample* t*-test were used to determine the association between perioperative characteristics and volume (high-volume versus low-volume). A *P* value of ≤0.05 was considered statistically significant. 5-year DFS and 5-year OS were estimated using the Kaplan-Meier method [21]. DFS was defined as time of surgery until recurrence of disease. OS was defined as the time of surgery until death. Multivariate Poisson Regression survival models were used to determine the effect of volume on DFS and OS. Variables in the univariate analysis that showed a significant association were then introduced into a Cox regression multivariate model.

## 3. Results

### 3.1. Study Population

Preoperative clinicopathological characteristics of the 453 low-volume and the 321 high-volume patients are shown in Table 1. The groups were comparable except the fact that a greater number of the low-volume patients had a higher ASA-classification (*P* < 0.001) and laparoscopic surgery was more frequently performed in the high-volume group compared to the low-volume group, 78% (*n* = 249) versus 59% (*n* = 266), respectively (*P* < 0.001). The type of resection also showed a difference (*P* < 0.001), largely caused by a higher number of abdominoperineal resections (APR) in the high-volume group. The significantly larger number of patients who received chemoradiotherapy as neoadjuvant regimen in this group can be explained by the higher number of rectal cancer cases (*P* < 0.001).

The intraoperative data are listed in Table 2. In the high-volume group, significantly less blood loss was observed compared to the low-volume group, 308 mL versus 547 mL, respectively (*P* < 0.001). Also the conversion rate in case of laparoscopic surgery was significantly lower in the high-volume group (18% versus 27%, *P* = 0.01). There was no significant difference in operative time between both groups.

The postoperative characteristics are listed in Table 3. A significantly more advanced tumor (T) stage (*P* = 0.03) and metastatic (M) stage (*P* < 0.001) were seen in the low-volume group. A larger median number of lymph nodes were harvested in the high-volume group, 15.3 versus 13.5 (*P* < 0.001). In the high-volume group the median postoperative hospital-stay was lower compared to the low-volume group: 10 versus 13 days, respectively (*P* < 0.001). No difference was seen between nodal (N) stages or resection margins and the number and neither did the severity of both surgical and nonsurgical adverse events show a difference in both groups.

### 3.2. Disease-Free Survival

**Figure 1:**
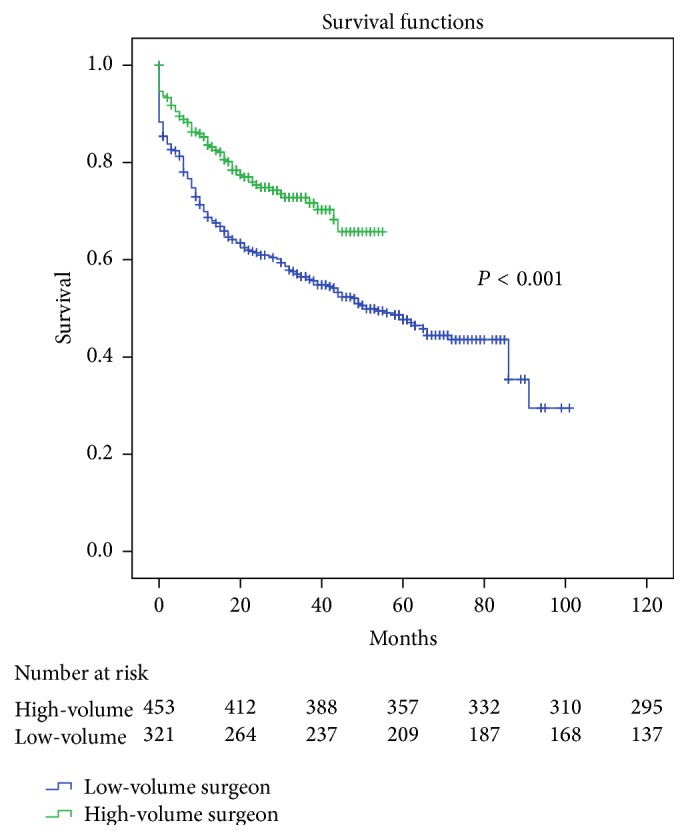
Kaplan-Meier curve for disease-free survival stratified for surgeon volume. Sixty-six percent of the patients operated on by a high-volume surgeon were free of disease after a median follow-up of four years, compared to 48% of those operated on by a low-volume surgeon (*P* < 0.001).

Median follow-up was four years. The 5-year DFS in the high-volume group was 66% compared to 48% in the low-volume group (*P* < 0.001, Figure 1).

We performed a univariate analysis to estimate the effect of all variables on the DFS. The high-volume group showed a significantly increased DFS (hazard ratio (HR) 0566; 95% CI 0.44–0.74; *P* < 0.001). The other pre-, intra-, and postoperative variables that showed a statistical significance for DFS in the univariate analysis are listed in the left half of Table 4. We then incorporated the statistically significant variables of the univariate analysis into a Cox multivariate regression model to determine which variables remained as prognostic factors for DFS. Surgeons' volume showed to be an independent prognostic factor for DFS in favor of the high-volume surgeon (HR 0.739; 95% CI 0.56–0.99; *P* = 0.04). Other independent prognostic factors for a longer DFS were lower patient's age (*P* < 0.001), lower ASA-classification (*P* = 0.05), and a lower T (*P* = 0.04), N (*P* < 0.001), and M (*P* < 0.001) stage. We also analyzed if the time periods (2004–2007 versus 2008–2011) had an influence on DFS; however, neither in the univariate or in the multivariate analysis did this show significance.

### 3.3. Overall Survival

**Figure 2:**
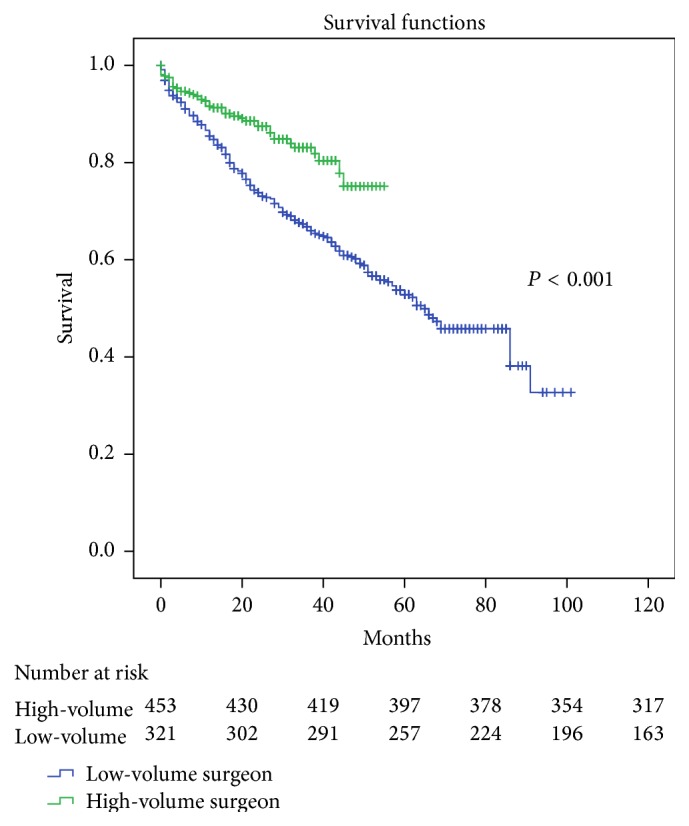
Kaplan-Meier curve for overall survival stratified for surgeon volume. Seventy-five percent of the patients operated on by a high-volume surgeon were alive after a median follow-up of four years, compared to 54% of those operated on by a low-volume surgeon (*P* < 0.001).

The patients in the high-volume group showed a 5-year OS of 75% as compared to 54% for the low-volume group (*P* < 0.001, Figure 2).

Similarly to what is described above, we performed a univariate analysis for OS. The high-volume surgeon was significantly associated with an increased OS (HR 0.495; 95%CI 0.35–0.69; *P* < 0.001). The other pre-, intra-, and postoperative variables that showed a statistical significance for OS in the univariate analysis are listed in the left half of Table 5.

After incorporating the statistically significant variables of the univariate analysis into a Cox multivariate regression model to determine which variables remained as prognostic factors for OS, the high-volume surgeon did not remain significant (HR 0.731; 95% CI 0.71–1.68; *P* = 0.09). We also analyzed if the time periods (2004–2007 versus 2008–2011) had an influence on OS; however, neither in the univariate or in the multivariate analysis did this show significance. The factors that did prove to be an independent prognostic factor were advanced age (*P* < 0.001), higher ASA-classification (*P* = 0.01), and higher N (*P* < 0.001) and M (*P* < 0.001) stage which showed to have an independent negative influence on OS. Laparoscopic surgery appeared to be a positive independent prognostic factor for OS (*P* < 0.001).

## 4. Discussion

In the current analysis we found that a high-volume surgeon is an independent prognostic factor for increased DFS for colorectal cancer surgery when compared to a low-volume surgeon. However, high-volume surgery did not remain as an independent prognostic factor for OS in the multivariate analysis. Although increased DFS is an important outcome in research, ultimately a longer OS is what is most desirable in medicine and what is important to the patient. Possibly in a larger cohort of patients we may show an increased OS in the future since OS did show to be significantly increased in the high-volume surgery patients in the univariate analysis.

Previous studies have been performed to investigate possible variables of short-term and long-term outcomes following colorectal resection for malignancies. These outcomes depend on numerous patient-, surgeon-, and hospital-related variables [5, 9, 22–27]. While the patient-related variables are difficult, if not impossible to adjust, efforts aimed at improving the perioperative care have been shown to have positive impact on the postoperative outcome. Some of these efforts include the administration of preoperative antibiotics, maintaining normothermia during surgery, and implementing an ERAS protocol [15, 16, 28–30]. Another approach that has been shown to be effective is implementing high-volume surgery of colorectal procedures [13]. Recent studies have shown an improved short-term outcome, when a high-volume surgeon performed the procedure [9, 23, 24, 31]. Studies reporting long-term effects for high-volume colorectal surgery, however, have shown less unanimous results [12, 26, 27, 32, 33].

Although high-volume surgery showed a significant relationship towards an increased OS in the univariate analysis, it did not remain as an independent prognostic factor for OS in the multivariate analysis. When looking at the difference in OS between the high-volume group and the low-volume group (Figure 2) in the univariate analysis, it seems likely that, with either a larger patient population or an increased median follow-up time, this observed difference could also become statistically significant in the multivariate analysis. This would of course be an important outcome for our patients, as increased OS is even more relevant than an increased DFS.

Our findings are in agreement with the outcomes of studies of low-volume surgical procedures, such as esophageal and pancreatic cancer surgery, in which it has been shown that the surgeon's caseload is an important predictor for outcome [34, 35]. Also support for our assumption of an increased OS can be found in the article by Rogers Jr. et al. who showed that, in a group of 26,644 patients with a median follow-up of 6 years, those who were operated upon by a high-volume surgeon had an increased OS following colorectal cancer [13]. The same is seen in the Cochrane analysis in which an improved survival is reported for both the high-volume surgeon and the high-volume hospital [6]. However, not all studies reporting survival after high-volume surgery for colorectal cancer are in agreement. Parry et al. and McArdle et al., for instance, reported no difference in OS. [27, 32, 33, 36, 37], showing that more research in this field is required before any definite statements can be made regarding the volume an individual surgeon should perform.

Laparoscopic surgery did turn out to be an independent prognostic factor for OS. Recently published literature has increasingly published a similar observation showing an improved OS following laparoscopic colorectal surgery when compared to open colorectal surgery [38–41]. It has been suggested that this improved OS observed in the recent years is mostly caused by the increased experience with the procedure together with technical and procedural advances.

High-volume surgery was associated with an increased DFS. Although this cannot be attributed to the implementation of high-volume surgeons alone, the fact that DFS remained as an independent prognostic factor in the multivariate analysis shows that high-volume surgery is attributed to improved survival. In a previous report, Renzulli et al. observed a similar increase in DFS for high-volume surgery [36].

Patients requiring an emergency procedure had a significantly worse DFS in the univariate analysis. However, in the multivariate analysis this did not remain significant showing that the difference in DFS seen between both groups cannot be explained by the timing of the surgery and the on-call surgeon.

Apart from the increased DFS in the multivariate and the increased OS in the univariate analysis, a number of perioperative variables also showed statistical significance in favor of the high-volume surgeon. In case a high-volume surgeon performed a laparoscopic procedure, a significantly lower number of conversions were observed. Furthermore, intraoperative blood loss was significantly less in the high-volume group and a greater number of lymph nodes were harvested leading to a more accurate staging. Previous studies have made similar observations [11, 42, 43]. A decrease in postoperative adverse events has been reported in case the operation was performed by a high-volume surgeon [9, 11]. However, similarly to Yasunaga et al. we did not observe such a decrease [4]. Possibly, underreporting of adverse events may have taken place in our study: the number of days a patient was admitted to the hospital following surgery by a high-volume surgeon was significantly lower, suggesting a quicker and uncomplicated recovery. Therefore, in our hospital, high-volume surgery does not only improve the DFS and possibly OS but also improve short-term outcome.

Some limitations of our report have to be addressed. The low-volume group consisted of more patients with a higher ASA-classification and a higher TNM-stage [16, 17]. The early drop of DFS seen in the low-volume patients that is demonstrated in the Kaplan-Meier curve is most likely due to the more advanced disease in this group (Figure 1). For this reason a multivariate analysis was conducted to correct for these differences in patient population. Even after this correction high-volume surgery remained as an independent factor for DFS. Rectal cancer resections were performed more frequently by the high-volume surgeons, which could have caused fewer postoperative complications due to increased experience. On the other hand, one could have expected more complications following the neoadjuvant radiotherapy and chemotherapy in this group, but this was not the case in the statistical analyses.

In conclusion, the current study shows that in our hospital high-volume surgery is an independent prognostic factor for increased DFS following surgery for colorectal cancer. Although high-volume surgery also significantly improved OS in the univariate analysis, it did not remain statistically significant in the multivariate analysis. It is possible that, with either more patients included or a longer follow-up time, this observed difference will also become statistically significant for OS. To our opinion, introducing high-volume surgeons will provide better perioperative care for patients suffering from colorectal cancer resulting in both improved short-term and long-term results.

## Figures and Tables

**Table 1:** Preoperative characteristics.

Characteristics	Low-volume surgeon (*n* = 453) Number of cases (%)	High-volume surgeon (*n* = 321) Number of cases (%)	*P* value
Gender			
Male	234 (52)	184 (57)	0.12
Female	219 (48)	137 (43)	

Age in years			
Median (95% CI)^e^	69 (46–92)	69 (48–90)	
<50	30 (7)	11 (3)	0.66
50–75	276 (61)	218 (68)	
>75	147 (32)	92 (29)	

ASA^a^-classification [16]			
1	101 (23)	57 (18)	**<0.001**
2	218 (47)	192 (60)	
3	112 (25)	69 (21)	
4	22 (5)	3 (1)	

Comorbidity			
No	147 (33)	102 (32)	0.83
Yes	305 (67)	219 (68)	
Cardiac			
No	367 (81)	251 (78)	0.30
Yes	85 (19)	70 (22)	
Pulmonary			
No	411 (91)	285 (89)	0.33
Yes	41 (9)	36 (11)	
Diabetes			
No	393 (87)	293 (91)	0.06
Yes	59 (13)	28 (9)	

BMI^b^			
Median (95% CI)^e^	26 (18.5–32.5)	26 (18.7–32.7)	0.54

Surgical technique			
Open	187 (41)	72 (22)	**<0.001**
Laparoscopic	266 (59)	249 (78)	

Type of resection			
Right colon	149 (33)	86 (27)	**<0.001**
Transversum	26 (6)	6 (2)	
Left colon	49 (11)	29 (9)	
Sigmoid	109 (24)	95 (29)	
LAR^c^	95 (21)	70 (22)	
APR^d^	25 (5)	35 (11)	

Neoadjuvant radiotherapy (rectum only)			
No	74 (56)	52 (45)	0.10
Yes	59 (44)	63 (55)	

Neoadjuvant chemoradiotherapy (rectum only)			
No	130 (98)	90 (78)	**<0.001**
Yes	3 (2)	25 (22)	

^a^American Society of Anesthesiology; ^b^body mass index; ^c^low anterior resection; ^d^abdominoperineal resection; ^e^95% confidence interval.

**Table 2:** Intraoperative characteristics.

Characteristics	Low-volume surgeon (*n* = 453) Number of cases (%)	High-volume surgeon (*n* = 321) Number of cases (%)	*P* value
Operative time			
Median in minutes (95% CI)^a^	148 (84–212)	146 (91–201)	0.66

Blood loss			
Median in mL (95% CI)^a^	547 (136–958)	308 (104–412)	**<0.001**

Conversion (laparoscopy only)			
No	195 (73)	205 (82)	**0.01**
Yes	71 (27)	44 (18)	

^a^95% confidence interval.

**Table 3:** Postoperative characteristics.

Characteristics	Low-volume surgeon (*n* = 453) Number of cases (%)	High-volume surgeon (*n* = 321) Number of cases (%)	*P* value
Tumor stage^a^			
1	34 (8)	22 (7)	**0.03**
2	82 (18)	74 (23)	
3	290 (64)	206 (65)	
4	45 (10)	15 (5)	

Nodal stage^a^			
0	250 (55)	197 (62)	0.18
1	140 (30.8)	78 (24)	
2	62 (14)	46 (14)	
3	1 (0.2)	0 (0)	

Metastatic stage^a^			
0	394 (87)	303 (94)	**<0.001**
1	59 (13)	18 (6)	

Number of lymph nodes			
Median (95% CI)^b^	13.5 (7.3–19.7)	15.3 (8.5–22.1)	**<0.001**

Resection margins			
Complete resection (R0)	430 (95)	314 (98)	0.11
Microscopically irradical (R1)	15 (3)	4 (1)	
Macroscopically irradical (R2)	8 (2)	3 (1)	

Length of hospital-stay			
Median in days (95% CI)^b^	13.1 (6.4–19.8)	10.2 (4.8–15.6)	**<0.001**

Surgical adverse events			
No	324 (72)	216 (67)	0.21
Yes	129 (28)	105 (33)	

Nonsurgical adverse events			
No	383 (85)	283 (88)	0.15
Yes	70 (15)	38 (12)	

Severity of adverse event			
No	277 (61)	197 (61)	0.74
Self-limiting	73 (16)	59 (18)	
Temporary, invasive procedure	87 (19)	57 (18)	
Lasting negative effect	2 (1)	2 (1)	
Death	14 (3)	6 (2)	

Reintervention			
None	364 (80)	266 (83)	0.20
Radiological	7 (2)	9 (3)	
Surgical	82 (18)	46 (14)	

Adjuvant chemotherapy			
No	313 (69)	243 (76)	**0.04**
Yes	140 (31)	78 (24)	

^a^According to the AJCC TNM-staging system [17]; ^b^95% confidence interval.

**Table 4:** Univariate and multivariate analysis of disease-free survival.

Characteristics	*N*	Univariate^a^			Multivariate		
		HR	95% CI	*P* value	HR	95% CI	*P* value
Low-volume surgeon	445^b^	1.000			1.000		
High-volume surgeon	316^b^	0.566	0.44–0.74	<0.001	0.736	0.55–0.98	**0.04**

Age in years				0.01			**<0.001**
<50	41	1.000			1.000		
50–75	486	1.052	0.61–1.80		1.261	0.67–2.37	
>75	234	1.304	1.16–1.49		2.034	1.01–4.08	

ASA-classification [16]				<0.001			**0.05**
1	158	1.000			1.000		
2	410	0.938	0.68–1.29		0.914	0.63–1.33	
3	181	1.547	1.09–2.19		1.301	0.86–1.97	
4	25	2.567	1.65–4.62		1.507	1.05–2.86	

Urogenital comorbidity	66	1.521	1.07–2.17	0.02	1.174	0.69–1.47	0.43

Open surgical technique	253	1.000			1.000		
Laparoscopic	508	0.518	0.41–0.65	<0.001	0.752	0.55–1.03	0.08

No conversion	396	1.000			1.000		
Conversion (laparoscopy only)	112	1.380	1.03–1.86	0.03	1.337	0.92–1.95	0.13

Elapsed time of the surgery	761	1.003	1.00–1.01	0.02	1.001	0.99–1.01	0.43

Blood loss intraoperatively	761	1.001	1.00–1.01	<0.001	1.000	0.99–1.00	0.20

Tumor stage^c^				<0.001			**0.04**
1	50	1.000			1.000		
2	153	1.293	0.63–2.67		1.307	0.59–2.91	
3	489	2.549	1.31–4.97		1.666	0.79–3.52	
4	58	6.609	3.21–13.6		2.814	1.21–6.54	

Nodal stage^c^				<0.001			**<0.001**
0	439	1.000			1.000		
1	216	2.484	1.91–3.23		1.769	1.28–2.46	
2	106	4.791	3.57–6.43		2.484	1.51–4.10	

Metastatic stage^c^				<0.001			**<0.001**
0	684	1.000			1.000		
1	77	9.697	7.29–12.9		7.093	4.99–10.1	

Positive lymph nodes	761	1.117	1.09–1.14	<0.001	1.031	0.98–1.08	0.21

Resection margins				<0.001			0.29
Complete resection (R0)	731	1.000			1.000		
Microscopically irradical (R1)	19	2.955	1.76–4.97		1.200	0.60–2.38	
Macroscopically irradical (R2)	11	3.080	1.58–6.00		0.551	0.23–1.32	

Length of hospital-stay	761	1.012	1.01–1.02	<0.001	1.004	0.99–1.01	0.39

No adverse event	656	1.000			1.000		
Adverse event	105	1.664	1.24–2.23	0.01	1.069	0.69–1.65	0.76

No adjuvant chemotherapy	546	1.000			1.000		
Adjuvant chemotherapy	215	2.104	1.67–2.65	<0.001	1.376	0.98–1.93	0.06

^a^Only significant factors listed; ^b^13 patients lost to follow-up, 8 low-volume patients, and 5 high-volume patients; ^c^according to the AJCC TNM-staging system [17].

**Table 5:** Univariate and multivariate analysis of overall survival.

Characteristics	*N*	Univariate^a^			Multivariate		
		HR	95% CI	*P* value	HR	95% CI	*P* value
Low-volume surgeon	451^b^	1.000			1.000		
High-volume surgeon	321	0.495	0.35–0.69	<0.001	0.731	0.51–1.04	0.09

Age in years				<0.001			**<0.001**
<50	41	1.000			1.000		
50–75	494	1.068	0.58–1.98		1.110	0.82–1.77	
>75	237	2.426	1.30–4.52		1.578	1.45–1.76	

ASA-classification [16]				<0.001			**0.01**
1	158	1.000			1.000		
2	410	1.384	0.92–2.08		1.340	0.83–2.16	
3	181	2.726	1.78–4.17		1.973	1.16–3.36	
4	25	5.398	3.07–9.49		3.136	1.54–6.41	

Comorbidity	523	1.509	1.13–2.01	<0.001	1.089	0.71–1.68	0.70
Cardiac	155	1.569	1.17–2.11	<0.001	1.190	0.79–1.79	0.40
Vascular	244	1.427	1.10–1.86	<0.001	1.084	0.78–1.52	0.64
Neurologic	56	1.663	1.09–2.54	0.02	1.190	0.92–2.41	0.10
Urogenital	66	1.749	1.20–2.54	<0.001	1.215	0.78–1.89	0.39

Open surgical technique	258	1.000			1.000		
Laparoscopic	514	0.522	0.41–0.67	<0.001	0.595	0.43–0.83	**<0.001**

No conversion	400	1.000			1.000		
Conversion (laparoscopy only)	114	1.510	1.10–2.08	0.01	1.418	0.93–2.16	0.10

Blood loss intraoperatively	772	1.001	1.00–1.01	<0.001	1.000	1.00–1.00	0.63

Tumor stage^c^				<0.001			0.67
1	50	1.000			1.000		
2	156	1.235	0.57–2.66		1.507	0.64–3.58	
3	495	1.999	0.98–2.66		1.435	0.64–3.23	
4	60	4.956	2.30–10.7		1.952	0.78–4.88	

Nodal stage^c^				<0.001			**<0.001**
0	445	1.000			1.000		
1	218	2.262	1.68–3.04		1.959	1.34–2.87	
2	108	4.997	3.61–6.92		3.091	1.77–5.40	
3	1	14.03	1.94–101		5.660	0.65–49.6	

Metastatic stage^c^				<0.001			**<0.001**
0	695	1.000			1.000		
1	77	5.020	3.73–6.76		3.883	1.34–2.87	

Number of positive lymph nodes	772	1.134	1.11–1.16	<0.001	1.042	0.99–1.10	0.13

Resection margins				<0.001			0.08
Complete resection (R0)	742	1.000			1.000		
Microscopically irradical (R1)	19	3.520	2.05–6.05		2.294	1.08–4.89	
Macroscopically irradical (R2)	11	4.153	2.12–8.12		1.798	0.71–4.53	

No adverse event	665	1.000			1.000		
Adverse event	107	1.938	1.42–2.65	<0.001	1.129	0.70–1.82	0.62

No adjuvant chemotherapy	554	1.000			1.000		
Adjuvant chemotherapy	218	1.517	1.17–2.98	<0.001	1.174	0.79–1.74	0.43

^a^Only significant factors listed; ^b^3 low-volume patients lost to follow-up; ^c^according to the AJCC TNM-staging system [17].
